# Comparison of pretreatment VMAT quality assurance with the integral quality monitor (IQM) and electronic portal imaging device (EPID)

**DOI:** 10.1002/acm2.13201

**Published:** 2021-02-17

**Authors:** Melissa Ghafarian, Michael Price, Manuel Morales‐Paliza

**Affiliations:** ^1^ Department of Radiation Oncology Vanderbilt University School of Medicine Nashville TN USA; ^2^ Department of Radiation Oncology Vanderbilt University Medical Center Nashville TN USA

**Keywords:** EPID, IQM, pretreatment VMAT QA

## Abstract

The purpose of this study was to compare pretreatment volumetric modulated arc therapy (VMAT) quality assurance (QA) measurements and evaluate the multileaf collimator (MLC) error sensitivity of two detectors: the integral quality monitor (IQM) system (iRT systems IQM) and the electronic portal imaging device (EPID) (Varian PortalVision aS1200). Pretreatment QA measurements were performed for 20 retrospective VMAT plans (53 arcs). A subset of ten plans (23 arcs) was used to investigate MLC error sensitivity of each device. Eight MLC error plans were created for each VMAT plan. The errors included systematic opening/closing (±0.25, ±0.50, ±0.75 mm) of the MLC and random positional errors (1 mm) for individual/groups of leaves. The IQM was evaluated using the percent error of the measured cumulative signal relative to the calculated signal. The EPID was evaluated using two methods: a novel percent error of the measured relative to the predicted cumulative signals, and gamma (γ) analysis (1%/1 mm, 2%/2 mm, 3%/3 mm and 3%/1 mm for Stereotactic Body Radiation Therapy plans). The average change in maximum dose obtained from dose‐volume histogram (DVH) data and change in detector signals for different systematic MLC shifts was also compared. Cumulative signal differences showed similar levels of agreement between measured and expected detector signals (IQM: 1.00 ± 0.55%; EPID: 1.22 ± 0.92%). Results from γ analysis lacked specificity. Only the 1%/1 mm criteria produced data with remarkable differences. A strong linear correlation was observed between IQM and EPID cumulative signal differences with MLC error magnitude (R = 0.99). Likewise, results indicate a strong correlation between the cumulative signal for both detectors and DVH dose (R_IQM_ = 0.99; R_EPID_ = 0.97). In conclusion, use of cumulative signal differences could be more useful for detecting errors in treatment delivery in EPID than γ analysis.

## 1 INTRODUCTION

Volumetric modulated arc therapy (VMAT), a form of rotational intensity‐modulated radiation therapy (IMRT), has been widely implemented in radiotherapy as a tool to deliver heterogeneous dose distributions providing high doses to target volumes while sparing normal tissues. In VMAT, multiple overlapping arcs with simultaneous variation of the gantry speed, multileaf collimator (MLC) movement, and dose rate are used to create fields with modulated beam intensities.^1^, ^2^, ^3^, ^4^ Despite its numerous advantages, the implementation of VMAT adds complexity to treatment planning and delivery thereby increasing the sources of error in their workflows.^5^, ^6^ These added uncertainties in the VMAT process highlight the need for patient specific pretreatment quality assurance (QA) of treatment plans to verify the accuracy of dose calculations and detect clinically relevant errors in radiation delivery.

Currently, most pretreatment QA for IMRT and VMAT plans is measurement based. A number of techniques can be used for measurements with the workflow typically consisting of recalculation of the approved treatment plan on a dosimeter and subsequent irradiation in the same geometry.^5^ The calculated and measured dose distributions are compared, and the plan is either approved or rejected for treatment based on in‐house specific acceptance criteria. An advancement in the science of QA for radiotherapy has been the development of Portal Dosimetry. The electronic portal imaging device (EPID) is a digital MV imaging detector attached opposite to the treatment head of the linac gantry. It is primarily used for verification of patient positioning during treatment; however, its dosimetric properties have led to its utilization in patient‐specific QA measurements.^7^, ^8^, ^9^, ^10^ This technique consists of comparing predicted portal dose distributions and acquired portal images using an evaluation method, typically the gamma (γ) index.^7^, ^11^ However, this methodology is intrinsically limited by numerous complications. Use of a portal dose prediction algorithm in place of the actual dose calculation algorithm is problematic and can mask errors, such as those related to the quality of the dose calculation model, that occur downstream from calculation of the fluence.^12^ Furthermore, EPID measurements are subject to issues from the use of digital detectors, such as contributions from electronic noise.^8^ Lastly, although the γ index is commonly employed in patient‐specific QA, limitations associated with its use should also be considered.^13^, ^14^

Recent advances in transmission detector technology offer a potential alternative to the EPID. The integral quality monitor (IQM) is a novel transmission detector consisting of a large‐area ionization chamber capable of performing high sensitivity charge collection measurements. The detector is mounted on the accessory tray of the linear accelerator treatment head and connects wirelessly to a controlling workstation via a Bluetooth transceiver.^15^ Patient‐specific VMAT QA can be performed by comparing chamber readings to calculated signal values.^16^ The IQM directly measures signal from the approved treatment plan generated with the actual dose calculation algorithm in the treatment planning system (TPS). The system is robust with fewer moving parts requiring less maintenance and Qualified Medical Physicist time allocation.^17^ Finally, IQM does not require use of the γ index for comparison of two‐dimensional dose distributions.

Previous studies in the literature have investigated the use of the IQM system in the context of pretreatment QA.^16^, ^18^, ^19^, ^20^ The study by Hoffman et al. showed that several types of IMRT errors can be found with the IQM system.^19^ Razinskas et al. investigated the use of the IQM system for VMAT verification which is inherently more complex.^16^ Saito et al. compared MLC error sensitivity of the IQM to other detectors commonly used for pretreatment VMAT QA.^20^ The aim of this study is to investigate the IQM as an alternative to Portal Dosimetry by comparing pretreatment VMAT QA results and evaluating the MLC error detection capabilities of the two systems.

## 2 MATERIALS AND METHODS

### 2.A Quality assurance devices

Pretreatment VMAT QA measurements were performed using the PortalVision aS1200 (Varian Medical Systems, Palo Alto, CA, USA) EPID and the transmission detector IQM (iRT Systems GmbH, Koblenz, Germany).

#### 2.A.1 Electronic portal imaging device

The PortalVision as1200 flat‐panel EPID used in this study consists of an amorphous silicon (a‐Si) photodiode detector array attached to a Varian TrueBeam (V2.7) linear accelerator gantry through a robotic arm. The detector features an active area measuring 40 cm × 40 cm with an 1190 × 1190‐pixel array with pixel pitch of 0.336 mm. Each pixel is composed of a metal plate covering a scintillator (Lanex Fast Back) over an a‐Si layer with embedded photodiode and thin film transistor on glass substrate.^21^, ^22^

EPID pretreatment QA of IMRT and VMAT plans consists of comparing acquired portal images to predicted portal dose images commonly using the γ index. Portal images are acquired by the detector measuring the fluence of each field and subsequently using analytic software to correlate the response to dose delivered.^23^ The relationship between the EPID readout signal per pixel and measured portal dose is determined by the imager calibration.^8^ The predicted portal dose distribution is calculated by the standalone algorithm portal dose image prediction (PDIP) (V13.6), which has an internal calculation resolution of 0.39 mm.^24^ Quantitative comparison of predicted and measured portal dose distributions is typically performed using the γ index developed by Low et al.^11^

#### 2.A.2 Integral quality monitor

The IQM detector is comprised of three aluminum alloy plates embedded in a guard ring and fixed into a polymethyl methacrylate (PMMA) frame housed in a carbon fiber cover. The two outer plates act as polarizing electrodes while the central plate acts as the collecting electrode. The outer electrodes are positioned at an angle relative to the central electrode. The PMMA frame acts as an insulator and also maintains the orientation and separation of the electrodes. The separation of the inner and outer electrodes increases linearly from 2 to 20 mm in the direction of MLC leaf motion creating a spatially sensitive detector response. Near the center of the chamber, the detector signal changes 0.5% per mm along the gradient. The active area of the detector is defined by the electrode surface area, which measures 26.5 cm × 26.5 cm, and covers a maximum field size of 40 cm × 40 cm at the radiation isocenter. During normal operation, the collecting and guard electrodes are held at ground potential and a potential bias of −500 V is applied to the high voltage outer electrodes. The detector features a wide dynamic range dual integrator electrometer and microprocessor. The dual integrator design of the electrometer prevents saturation that typically occurs with large charge measurements while maintaining the accuracy and resolution of low charge measurements.^25^

During measurements, the radiation beam passes through the detector and the total charge produced by photon interactions in the active volume is collected by the ion chamber and reported by the IQM Monitor Software (V1.6.7) in real time.^15^, ^25^ For VMAT fields, the software reports the signal for individual beam segments and the cumulative signal for the entire field. Live monitoring of the gantry and collimator angles is performed by the integrated inclinometer. The raw measurement is corrected for temperature and pressure variations, chamber positional sensitivity, and field size effects and reported in arbitrary units (counts).^25^

Patient‐specific VMAT QA can be performed using the IQM system by evaluating agreement between the expected and measured signals for a given treatment field.^16^ This can be done in real time or after the field has been delivered.

The IQM Calc Software (V1.6.7) calculates the predicted signal for each beam segment (i.e., control point) by using field parameters including jaw positions, MLC leaf positions, and monitor units (MU), obtained from the DICOM‐RT Plan file. The algorithm uses a beam model consisting of two radiation sources to account for signal contributions from both open regions of the field and attenuated regions of the field passing through the beam's collimating elements. For a beam segment of U MU, the signal (C_IQM_) across the area of the chamber is given by

(1) $$C_{\text{IQM}}=U\cdot \text{AOF}\left(x,y\right)\cdot \frac{N_{\text{IQM}}}{n\times m}\cdot \sum_{i,j}^{n,m}S_{\text{IQM}}\left(i,j\right)\left[\left(1‐f_{s}\right)I_{p}\left(i,j\right)+f_{s}I_{s}\left(i,j\right)\right]$$

where (i,j) are positional indices in an n x m calculation array. Signal variations over the calculation array are accounted for by the terms under summation. S_IQM_ accounts for chamber positional sensitivity. I_p_ and I_s_ are the relative contributions of the primary and secondary fluence intensities, respectively, which are adjusted by the relative secondary fluence strength parameter, f_s_. AOF is the area output factor used to adjust for machine output variations due to field size (x,y). N_IQM_ normalizes the signal to the chamber‐specific response for a 10 x 10 cm^2^ reference field size.^15^, ^25^

### 2.B Treatment plan selection and delivery

Twenty retrospective VMAT plans (53 arcs) were selected for patient‐specific QA measurements based on anatomical treatment site. The plans included: seven head and neck plans, five chest plans, two stereotactic body radiation therapy (SBRT) spine plans, and six pelvic plans. Three of the chest plans were lung SBRT plans and three of the pelvic plans were prostate plans. Each plan consisted of at least two arcs. The treatment plans were created using the Eclipse (V13.6, Varian Medical Systems, Palo Alto, CA, USA) TPS. Dose calculations were performed using the anisotropic analytical algorithm (AAA) with a 0.15 cm grid size. The treatment plans were delivered on a TrueBeam linear accelerator (V2.5, Varian Medical Systems, Palo Alto, CA, USA) equipped with a Millennium 120 MLC using the 6 MV nominal photon beam energy.

A subset of ten VMAT plans (23 arcs) was selected to compare detector sensitivity to MLC errors. Plans selected for this part of the study had a > 95% γ passing rate with 3%/3 mm criteria. The plans included: three head and neck plans, two lung SBRT plans, three prostate plans, and two spine SBRT plans. Eight types of MLC error plans were created for each plan: systematic opening (0.25, 0.50, and 0.75 mm) and closing (−0.25, −0.50, −0.75 mm) of the MLC, and random 1.00 mm shifts applied to every fourth leaf in bank A as well as groups of five leaves in bank B. A MATLAB program developed in‐house was used to introduce errors in the clinical treatment plans.

### 2.C Evaluation metrics

Agreement between expected and measured values for the two systems was compared using the cumulative signal, γ index, and dose‐volume histogram (DVH) data. The γ index is not an appropriate tool for evaluation of IQM measurements as the system does not provide a two‐dimensional spatial response. Hence, a novel evaluation metric, the EPID cumulative signal (C_EPID_), was developed in addition to the γ index to directly compare the two systems. A comparison of detector response to DVH data was also performed for plans with MLC errors.

#### 2.C.1 Cumulative signal

The IQM and EPID were evaluated using the cumulative signal difference (CSD) given by the deviation of the measured cumulative signal (C_meas_) relative to the reference cumulative signal (C_ref_) for each field:

(2) $$\text{CSD}\left[\%\right]=\left(\frac{C_{\text{meas}}‐C_{\text{ref}}}{C_{\text{ref}}}\right)\times 100$$

In this equation, C_ref_ is the expected signal calculated by each system. A smaller CSD indicates less deviation of the measured detector signal (C_meas_) from its expected value (C_ref_). For the IQM, the value of C_ref_ is calculated by the IQM Calc module and equivalent to the sum of the signal contributions from all individual beam segments shown in Eq. (1). For EPID measurements, cumulative signal values, C_ref_ and C_meas_, were derived from the predicted (PDIP) and measured portal images, respectively, by summing individual pixel values (I_i,j_) over the 1190 × 1190 array:

(3) $$C_{\text{EPID}}=\sum_{i=1,j=1}^{1190,1190}I_{\text{i,j}}$$

A lower threshold equivalent to 1% of the maximum pixel value was applied to remove signal contributions from electronic noise. This threshold value was found to minimize noise and improve the signal‐to‐noise ratio. Lower threshold values did not remove enough of the signal noise, while higher threshold values eliminated too much data from the analysis.

Detector error sensitivity (S_error_) was also evaluated using the cumulative signal difference, but with the reference value (C_ref_) substituted by measured cumulative signal of the original plan (C_meas,original_):

(4) $$S_{\text{error}}\left[\%\right]=\left(\frac{C_{\text{meas},\text{error}}‐C_{\text{meas},\text{original}}}{C_{\text{meas},\text{original}}}\right)\times 100$$

In Eq. (4), C_meas,error_ is the cumulative signal measured for the field with the MLC error.

#### 2.C.2 γ analysis

Portal images were further evaluated using γ analysis to provide a comparison to current methodology.^11^ The improved γ algorithm in the ARIA RTM Portal Dosimetry application (V13.6, Varian Medical Systems, Palo Alto, CA, USA) was used to calculate γ passing rates for each field.^11^, ^26^ Evaluation was performed using global normalization and absolute dose. The region of interest (ROI) was defined by the maximum MLC opening plus 0.5 cm. The dose threshold was set to 10% to exclude low‐dose areas with minimal clinical relevance which could significantly bias results.^5^ The following dose difference (DD) and distance‐to‐agreement (DTA) criteria were used, DD/DTA: 3%/3, 2%/2, and 1%/1 mm. SBRT plans were also evaluated with the 3%/1 mm criteria.

#### 2.C.3 DVH comparison

Correlation between detector response and treatment plan dose was evaluated for MLC error plans. Maximum doses for target volumes and organs at risk were obtained from DVH data from the original and error plans in the TPS. The average relative change in dose caused by each error was calculated and compared to the IQM and EPID cumulative signal difference.

#### 2.C.4 Effects of different field parameters on detector response

The following field parameters were evaluated to determine their effect on IQM and EPID detector response: modulation index (MI) and field size. The MI indicates the degree of modulation for each arc and was defined as the ratio of MU to dose (cGy) as specified by the TPS. The field size was defined by the opening of the collimator jaws. For arcs with jaw tracking, the field size was defined by the maximum opening of the jaws throughout the arc.

### 2.D Statistical analysis

Linear correlations between different variables reported were evaluated using the Pearson correlation coefficient (R). The correlation has a value between +1 and −1, where +1 indicates a total positive linear correlation and −1 indicates a total negative linear correlation. To determine whether the correlation was statistically significant, the probability (*p*‐) value was calculated and compared to a significance level (α) of 0.01. Correlations with *p*‐values ≤ α were determined to have a correlation that is different from 0 and statistically significant within a 99% confidence interval.

## 3 RESULTS

### 3.A Pretreatment VMAT QA

Evaluation of the cumulative signal showed agreement between the measured and calculated reference signal with values symmetrically distributed around the mean value (±standard deviation). For all 20 VMAT plans (50 arcs) — with the exception of three arcs with CSD values in the 95th percentile — agreement with the reference signal was within 1.00% (±0.55) and 1.22% (±0.92) for the IQM and EPID, respectively. A summary of the distribution of CSD values for IQM and EPID measurements is provided in Table 1. Excluding outliers, 98% of fields measured with the IQM system were within ±2% of calculated values, whereas only 82% of fields measured with EPID were within ±2% of their predicted values. No statistically significant correlation was observed between the CSD values for the two detectors. Measurement reproducibility variation was 0.14% for the IQM and 0.37% for the EPID. Cumulative signal stability change over a 9‐month period was 0.22% for the IQM and 0.87% for the EPID.

**TABLE 1:** Cumulative signal differences (CSD) and γ passing rate frequency distribution for 20 VMAT plans (n = 50 arcs).

CSD (%)	Frequency (No. of fields/cumulative %)												
	IQM		EPID		γ passing rate (%)	3%/3 mm		2%/2 mm		1%/1 mm		3%/1 mm^a^	
≤1	27	54	24	48	≥98	50	100	49	98	11	22	12	100
1–2	22	98	17	82	95–98	0	100	1	100	15	52	0	100
2–3	1	100	5	92	90–95	0	100	0	100	9	70	0	100
≥3	0	100	4	100	≤90	0	100	0	100	15	100	0	100

^a^ Criteria used for fields from SBRT plans.

**FIG. 1:**
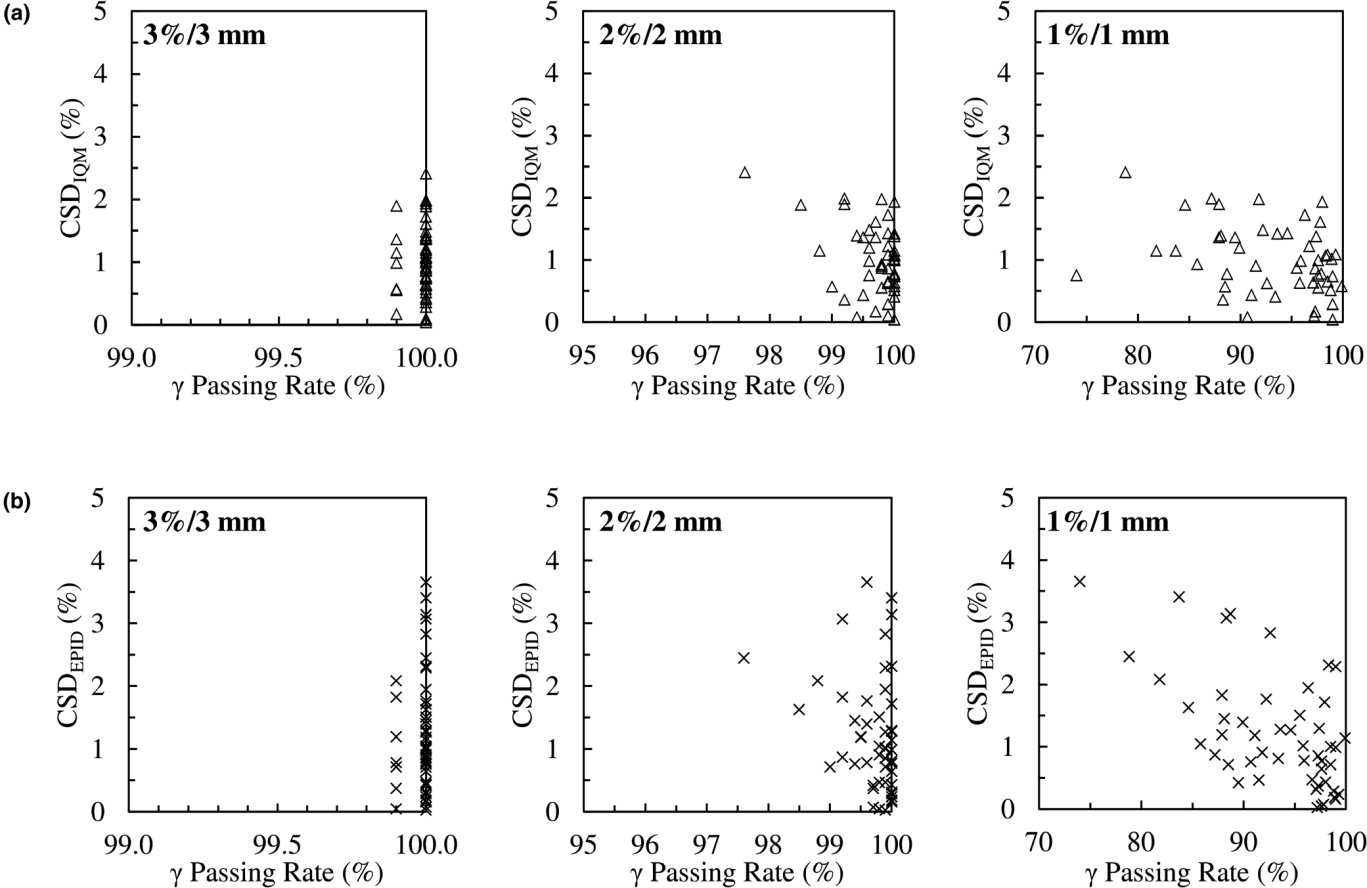
(a) IQM cumulative signal differences vs γ passing rates, (b) EPID cumulative signal differences vs. γ passing rates for 20 retrospective VMAT plans (n = 50 arcs).

Average γ passing rates for portal images evaluated using the 3%/3, 2%/2, and 1%/1 mm criteria were 99.99% (±0.03), 99.70% (±0.45), and 93.17% (±5.88), respectively. Passing rates for fields from SBRT plans evaluated using the 3%/1 mm criteria had a mean value of 99.86% (±0.26). Figure 1 shows IQM and EPID cumulative signal differences vs γ passing rates for all fields. Moderate negative correlations were observed comparing IQM and EPID cumulative signal deviations with γ passing rates for the 2%/2 mm (R_IQM_ = −0.41, R_EPID_ = −0.26) and 1%/1 mm criteria (R_IQM_ = −0.33, R_EPID_ = −0.26).

Mean CSD values and γ passing rates for different treatment sites are provided in Table 2. No correlation was observed between IQM and EPID cumulative signal differences with respect to plan type. In some instances, such as chest plan measurements, plans having the highest levels of agreement when measured using the IQM, were found to exhibit the greatest deviation from the calculated reference signal when measured using the EPID. Furthermore, there was no visible correlation between γ passing rates and IQM or EPID cumulative signals when evaluated by plan type.

**TABLE 2:** IQM and EPID pretreatment VMAT QA results by treatment site. Cumulative signal differences (CSD) and γ passing rates are presented (n = 50 arcs).

Plan type	CSD (%, mean ± SD)		γ passing rate (%, mean ± SD)			
	IQM	EPID	3%/3 mm	2%/2 mm	1%/1 mm	3%/1 mm^a^
Chest	0.81 ± 0.46	1.59 ± 1.19	100.00 ± 0.00	99.94 ± 0.12	93.63 ± 7.83	
└ Chest (other)	0.56 ± 0.48	2.08 ± 1.82	100.00 ± 0.00	99.90 ± 0.17	88.52 ± 10.24	
└ Lung (SBRT)	0.99 ± 0.39	1.24 ± 0.25	100.00 ± 0.00	99.97 ± 0.05	97.27 ± 2.33	100.00 ± 0.00
Head and Neck	0.99 ± 0.62	0.93 ± 0.79	99.98 ± 0.04	99.59 ± 0.57	93.79 ± 5.31	
Pelvis	1.20 ± 0.51	1.17 ± 0.56	99.98 ± 0.04	99.59 ± 0.46	91.52 ± 5.62	
└ Pelvis (other)	1.18 ± 0.54	1.40 ± 0.40	99.97 ± 0.05	99.37 ± 0.47	87.83 ± 3.48	
└ Prostate	1.23 ± 0.53	0.81 ± 0.62	100.00 ± 0.00	99.93 ± 0.08	97.05 ± 2.78	
Spine (SBRT)	0.90 ± 0.61	1.57 ± 1.28	100.00 ± 0.00	99.80 ± 0.34	94.76 ± 4.23	99.66 ± 0.36

^a^ Criteria used for fields from SBRT plans.

The effects of different field parameters on detector response were evaluated. A moderate positive correlation (R = 0.50) was observed between the IQM cumulative signal difference and MI. There was no evidence indicating EPID cumulative signal differences were affected by the degree of field modulation. Additionally, field size and MU were not found to be statistically significant predictors of the cumulative signal difference for the detectors. Although, there was a visible negative correlation between γ passing rates and field size with coefficients of R = −0.24, R = −0.47, and R = −0.58 for the 3%/3, 2%/2, and 1%/1 mm criteria, respectively.

### 3.B MLC error sensitivity

**FIG. 2:**
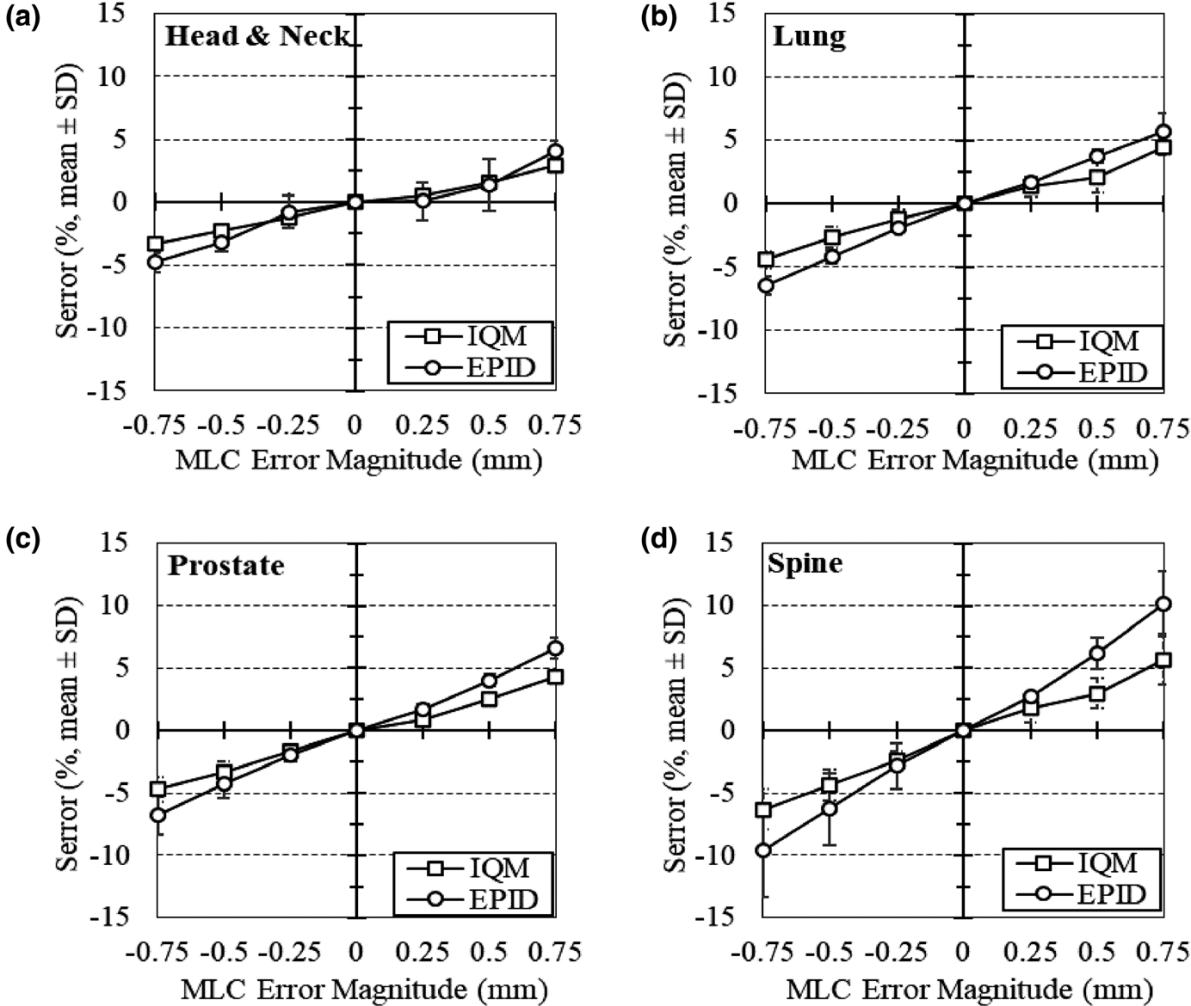
IQM and EPID detector error sensitivity (S_error_) by MLC error magnitude and plan type. The results for (a) Head and Neck, (b) Lung (SBRT), (c) Prostate, and (d) Spine (SBRT) plans are represented. Values shown are measured cumulative signal differences for error plans relative to the original treatment plan.

In the next step, ten of the clinical VMAT plans (23 arcs) were modified to include MLC errors and evaluated in the same way. Table 3 summarizes detector error sensitivity (S_error_) and Portal Dosimetry γ passing rates by MLC error type and magnitude. A strong correlation was observed between detector response and the magnitudes of systematic MLC errors. Correlation coefficients for the IQM and EPID cumulative signal and MLC shift magnitude are R = 0.99 and R = 0.99, respectively. On average, γ passing rates decreased as the magnitude of the shift increased. In particular, the strictest DD/DTA criteria of 1%/1 mm, were most sensitive to MLC errors (R = −0.55). Furthermore, systematic errors produced greater changes in signal/passing rate than random errors. However, changes in signal for open/closed errors were not equivalent. Systematic closed errors resulted in slightly greater deviations from the clinical plan compared to systematic open errors of the same magnitude. This effect was most noticeable in evaluation of the EPID measurements, particularly γ analysis. Figure 2 depicts the S_error_ for both detectors as a function of MLC systematic error for different treatment sites.

**TABLE 3:** Change in DVH maximum dose (ΔD_max_), detector error sensitivity (S_error_) and Portal Dosimetry γ passing rates for ten VMAT plans (n = 23 arcs) by MLC error type.

MLC error		ΔD_max_	S_error_ (%, mean ± SD)		γ Passing Rate (%, mean ± SD)		
Type	Magnitude (mm)	(%, mean ± SD)	IQM	EPID	3%/3 mm	2%/2 mm	1%/1 mm
Systematic Close	−0.75	−3.66 ± 2.33	−4.52 ± 1.48	−6.65 ± 2.67	98.96 ± 1.19	94.33 ± 3.92	65.40 ± 12.56
	−0.50	−2.48 ± 1.64	−3.04 ± 1.12	−4.34 ± 1.89	99.63 ± 0.52	97.57 ± 2.10	80.01 ± 8.54
	−0.25	−1.24 ± 0.90	−1.57 ± 0.64	−1.73 ± 1.41	99.92 ± 0.17	99.40 ± 0.76	91.41 ± 5.53
Systematic Open	0.25	1.44 ± 1.05	1.01 ± 0.83	1.36 ± 1.37	99.98 ± 0.05	99.85 ± 0.18	96.71 ± 2.68
	0.50	2.87 ± 1.98	2.22 ± 0.97	3.52 ± 2.28	99.88 ± 0.26	99.43 ± 0.84	93.19 ± 5.26
	0.75	4.30 ± 2.96	4.15 ± 1.48	6.34 ± 2.69	99.30 ± 1.29	97.50 ± 3.45	80.03 ± 10.73
Every fourth leaf (Bank A)	1.00	0.84 ± 0.43	0.86 ± 0.48	1.20 ± 1.03	99.96 ± 0.08	99.60 ± 0.65	94.20 ± 3.68
Groups of 5 leaves (Bank B)	1.00	1.34 ± 1.09	1.59 ± 0.90	2.61 ± 1.46	99.84 ± 0.30	99.26 ± 0.99	92.17 ± 3.51

**FIG. 3:**
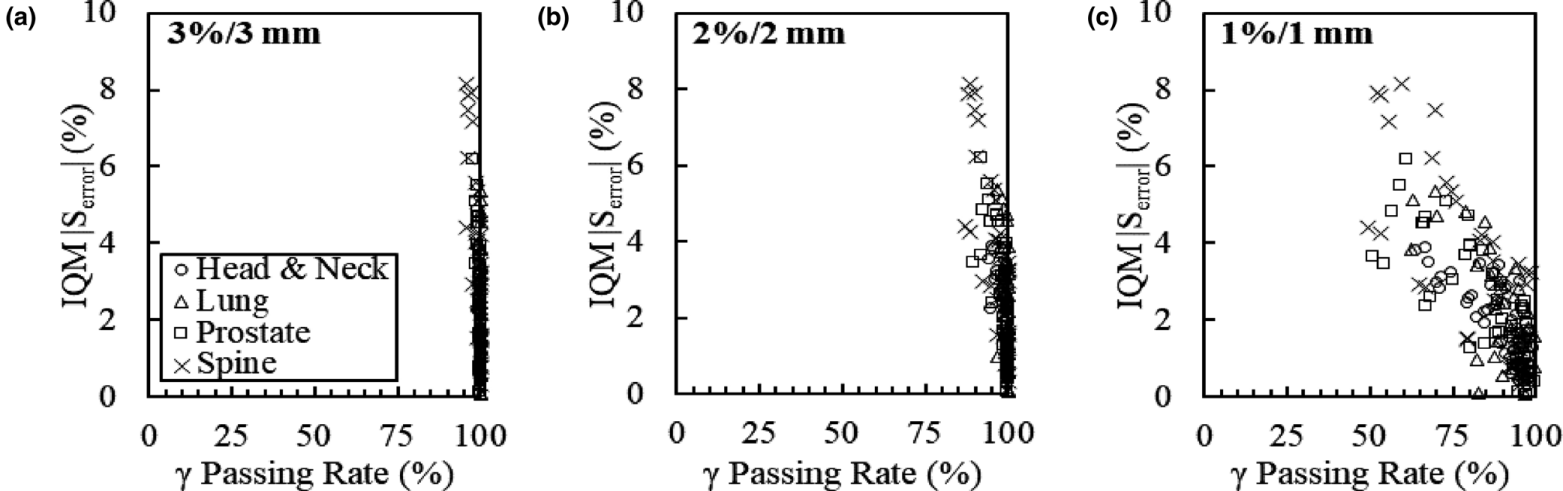
IQM error sensitivity (|S_error_|) and corresponding γ passing rates for all MLC errors by plan type. Results for head and neck, lung, prostate, and spine plans evaluated using the (a) 3%/3 mm, (b) 2%/2 mm, (c) and 1%/1 mm criteria are presented. Cumulative signal differences shown are for the error plan relative to the clinical plan.

There was a visible correlation between the IQM cumulative signal and γ passing rates for the error plans. Correlation coefficients of the cumulative signals and 3%/3, 2%/2, and 1%/1 mm γ passing rates are R = −0.68, R = −0.73, and R = −0.77, respectively. Figure 3 illustrates the relationship between IQM cumulative signal differences in altered plans and corresponding γ passing rates by treatment site.

Detector error sensitivity statistics for the cumulative signal and γ index are provided in Table 4. Based on measured signal stabilities determined in the first part of this study, tolerance and action limits for detection of MLC errors using the cumulative signal were defined as two and three standard deviations from the mean CSD. Thus, S_error_ tolerance and action limits were 2σ = 1.10 % and 3σ = 1.65% for the IQM, and 2σ = 1.84% and 3σ = 2.76% for portal measurements. Based on this approach, the following results were observed in the evaluation of IQM and EPID measured cumulative signal differences. Approximately 74% of fields measured with the IQM were within watch limits and 57% failed to meet acceptance criteria. The rate of error detection was slightly lower in EPID measurements where approximately 70% of fields were within watch limits and 52% fails to meet acceptance criteria. The IQM had a higher or equivalent rate of detection than EPID for six of the eight error types. Tolerance and action limits used for evaluating EPID γ passing rates were ≤95% and ≤90%, respectively. The γ index did not detect any of the errors in the modified plans when the 3%/3 mm criteria were used. Moreover, results from analysis using the 2%/2 mm criteria showed that passing rates did not meet tolerance and action limits for 11% and 3% of fields, respectively. Results from the 1%/1 mm analysis showed at least one field failing to meet the action limit for each type of error. For these criteria, approximately 68% of fields were within watch limits and 46% of fields failed to meet the ≥90% acceptance criteria.

**TABLE 4:** MLC errors detected using the cumulative signal (S_error_) and γ analysis for ten VMAT plans.

MLC error		|S_error_| > 2σ (No. of fields)		|S_error_| > 3σ (No. of fields)		γ passing rate ≤ 95% (No. of fields)			γ passing rate ≤ 90% (No. of fields)		
Type	Magnitude (mm)	IQM	EPID	IQM	EPID	3%/3 mm	2%/2 mm	1%/1 mm	3%/3 mm	2%/2 mm	1%/1 mm
Systematic close	−0.75	23	23	23	23	–	11	22	–	4	21
	−0.50	23	23	22	18	–	4	22	–	–	20
	−0.25	20	9	8	4	–	–	17	–	–	7
Systematic open	0.25	6	8	4	3	–	–	4	–	–	1
	0.50	22	21	14	17	–	–	10	–	–	7
	0.75	23	23	23	22	–	5	22	–	2	19
Every fourth leaf (Bank A)	1.00	4	7	2	–	–	–	11	–	–	3
Groups of 5 leaves (Bank B)	1.00	15	14	8	9	–	–	17	–	–	7
Total (n = 184):		136	128	104	96	0	20	125	0	6	85

**FIG. 4:**
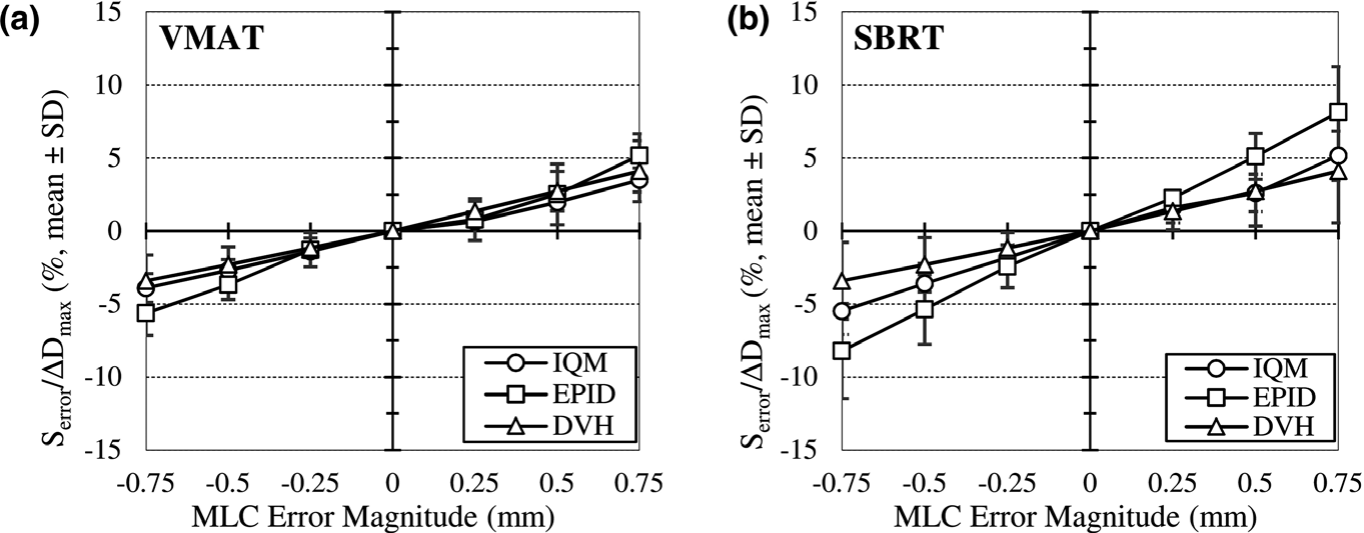
Average change in IQM and EPID detector response (S_error_) and change in DVH maximum dose (ΔD_max_) to target volumes and organs at risk due to systematic open/close MLC errors for (a) VMAT and (b) SBRT treatment plans.

Lastly, the relationship between dosimetric data from the TPS and detector response to MLC errors was investigated. IQM and EPID cumulative signal differences and changes in maximum dose are provided in Table 3. Values for VMAT and SBRT treatment modalities are depicted in Fig. 4. IQM and EPID signal changes have similar rates of agreements with respect to changes in dose for VMAT plans. However, EPID signal changes had a larger deviation from DVH values in SBRT plans, which tend to have smaller field sizes. While a strong correlation was observed in all field measurements using both detectors, IQM values were closer to dose values in the TPS (R_IQM_ = 0.99; R_EPID_ = 0.97).

## 4 DISCUSSION

Pretreatment VMAT QA measurements are needed to ensure the fidelity of radiation treatment delivery. The efficacy of a QA program is subject to the accuracy and reliability of the measurement methods and evaluation techniques used. Portal Dosimetry has been widely adopted as a QA technique with advantages and limitations of the technology reported in the literature.^10^ The question this manuscript aims to answer is whether the IQM system can improve upon the limitations of Portal Dosimetry and be clinically implemented as a new tool for performing pretreatment VMAT QA measurements. This study has led to three main findings as detailed below.

First, the IQM signal calculation was found to be highly accurate when used as a reference quantity for pretreatment VMAT QA. The calculation module was validated based on cumulative signal differences and γ analysis. The average IQM signal deviation for 20 clinical VMAT plans was 1.00%. This result compares well with the value of 0.96% reported in the study by Pasler et al. for 15 VMAT plans.^27^ Similarly, the data in this study are in agreement with the cumulative signal deviation of −0.48% reported by Razinskas et al. for 30 VMAT plans.^16^ It is also comparable to the newly developed average EPID cumulative signal deviation of 1.22% obtained in this study. Correlation between IQM signal deviation and EPID γ passing rates helped to further confirm accuracy of the IQM signal calculation. While these findings show that the cumulative signal difference is an acceptable metric for evaluation of pretreatment VMAT QA measurements, future studies may also find it useful to investigate alternative methods to achieve a higher sensitivity, such as the use of running averages of three to five segment signals in addition to the cumulative signal as proposed by Razinskas et al.^16^

Second, results indicate that the IQM has error detection capabilities useful in the context of pretreatment VMAT QA. Cumulative signal analysis showed that both detectors were sensitive to small shifts in MLC leaf position. IQM signal deviations were linearly correlated (R = 0.99) with MLC shifts. On average, systematic errors resulted in a 1% to 4% change in signal. Somewhat larger deviations were observed in the response of both detectors for fields in which a systematic closing of the MLC was applied (Table 3). This may be attributed to the decrease in signal‐to‐noise ratio as the subfields are reduced, which are not properly accounted for, especially by EPID. Use of tolerance and action limits revealed the superior error detection capabilities of the IQM system (Table 4). This was particularly true when results were compared to those of Portal Dosimetry γ analysis in which most errors were not detected unless the strictest criteria of 1%/1 mm were applied. It was also observed that IQM measurements had greater reproducibility and detector signal stability than the EPID. Further comparison with DVH data from the TPS showed that cumulative signal changes due to MLC errors were correlated with similar changes in dose to the patient. While this relationship was observed in both detectors, the correlation to dose was visibly stronger with the IQM, as revealed in Fig. 4. This result could lead to use of the detector for additional applications, such as in the estimation of dose to the patient.

Third, results reported in this study highlight the limitations associated with using Portal Dosimetry and the γ index. EPID cumulative signal and γ passing rates were subject to larger variations than the IQM. Evaluation of the EPID using the cumulative signal required application of a lower threshold to account for contributions from electronic noise. Given the unpredictable nature of electronic noise, choosing an appropriate threshold can be difficult. A threshold of 1% of the maximum pixel value was chosen for this study as it adequately removed noise contributions across all plans without substantially lowering the signal‐to‐noise ratio. Additionally, loss of detector panel calibration can lead to false positives in evaluation of QA results and require periodic detector panel recalibration. These limitations do not apply to the IQM system and directly affect EPID measurement reproducibility and signal stability having noticeable impacts on VMAT QA results. Lastly, the γ index was not useful in detecting most MLC errors when the 3%/3 and 2%/2 mm criteria were used. Evaluation of EPID data with the γ index (Table 2) showed that all fields produced similar pass rates for the 3%/3 and 2%/2 mm criteria and remarkable differences were only observed using the 1%/1 mm criteria. This finding illuminates the lack of specificity in results when using the γ index with the EPID and underscores the need for further evaluation with a different method, such as the PTW Octavius phantom inserted in the 2D‐array.^20^

Another point of discussion relating to the IQM for pretreatment VMAT QA is the clinical implementation of this technology. First, use of an auxiliary device like the IQM requires the purchase of additional hardware and software that are not required for performing pretreatment VMAT QA with the EPID, a system that is already included on most modern linacs. Additionally, clinical procedures must be established in the event that VMAT QA is performed with the IQM and a plan does not meet the action limit. An obvious drawback of the IQM cumulative signal is that it cannot provide additional information regarding the dose distribution required to diagnose the issue. In these cases, it may be helpful to look at the signal for individual segments within the field or to use a secondary device, such as the EPID itself, IBA MatriXX with COMPASS, Scandidos Delta4, or SunNuclear ArcCHECK, among others discussed in other studies, for further investigation.^16^, ^18^, ^20^

## 5 CONCLUSIONS

This study compared IQM and EPID detector performance in the context of pretreatment VMAT QA. While the IQM system does not provide a two‐dimensional spatial dose distribution, the transmission detector improves upon some of the limitations associated with Portal Dosimetry and γ analysis. The IQM detector demonstrated higher reproducibility and signal stability than EPID. The IQM system was also more sensitive to MLC errors than the traditional Portal Dosimetry technique utilizing γ analysis. Although the detector error sensitivity based on measured cumulative signal differences correlated well with dosimetric data obtained from the TPS for both detectors, the IQM signal presented a stronger correlation. Scrutiny of the data reveals that detecting errors in treatment delivery by EPID could be better attained by calculating cumulative signal differences in lieu of performing γ analysis. Clinical implementation of the IQM system may involve using it as a primary measurement device, and utilizing the EPID or other secondary QA device for finer diagnostics.

## CONFLICT OF INTEREST

No conflicts to disclose.

## AUTHOR CONTRIBUTION STATEMENT

All authors satisfy the authorship requirements and do not have anything to disclose.
